# Phenotypic and Phylogenetic Characterization of Cu Homeostasis among *Xylella fastidiosa* Strains

**DOI:** 10.3390/pathogens10040495

**Published:** 2021-04-20

**Authors:** Qing Ge, Ranlin Liu, Paul A. Cobine, Neha Potnis, Leonardo De La Fuente

**Affiliations:** 1 Department of Entomology and Plant Pathology, Auburn University, Auburn, AL 36849, USA; qzg0007@auburn.edu (Q.G.); rzl0060@auburn.edu (R.L.); nzp0024@auburn.edu (N.P.); 2 Department of Biological Sciences, Auburn University, Auburn, AL 36849, USA; pac0006@auburn.edu

**Keywords:** Cu homeostasis, *Xylella fastidiosa*, Cu-related genes, phylogenetic tree, Cu MIC

## Abstract

*Xylella fastidiosa* is a bacterial pathogen causing severe diseases and asymptomatic colonization in more than 600 plants worldwide. Copper (Cu) is a widely used antimicrobial treatment for various plant diseases, including those affecting *X. fastidiosa* hosts. Cu homeostasis among *X. fastidiosa* strains from different geographical locations and host species has not been characterized. Here, we assessed minimum inhibitory concentration (MIC) of Cu for 54 *X. fastidiosa* strains. We observed strain-level variation in MIC values within each subspecies. We hypothesized that these differences could be explained by sequence variation in Cu homeostasis genes. Phylogenies based on *copA*, *copB*, *copL*, and *cutC* were created using 74 genomes (including 43 strains used in vitro) of *X. fastidiosa*, showing that the phylogenetic clustering of Cu homeostasis associated with clustering was based on core genome phylogenies, rather than on pattern of MIC. No association was found among Cu MIC, subspecies classification, and host and location of isolation, probably due to uneven and limited group of strains whose genomes are available. Further analysis focused on a subgroup of isolates from Georgia’s vineyards that shared similar Cu-related phenotypes. Further research is needed to better understand the distribution of Cu homeostasis for this pathogen.

## 1. Introduction

*Xylella fastidiosa*, a gram-negative bacterial pathogen, can colonize and infect ~600 different host species, including agricultural crops, landscape trees, and weeds [1,2]. The pathogen has a wide geographical distribution; it has been reported in many countries such as the U.S., Brazil, Italy, and Spain, among others [3,4,5,6]. The spread of *X. fastidiosa* causes great damage to agricultural production and has a significant economic impact. Just in California (CA, U.S.), it results in the grape industry losses higher than $100 million every year [7]. The spread of *X. fastidiosa* in Europe would lead to future economic impact in the billions of Euros [8]. A study of *X. fastidiosa* isolates from different locations in CA suggested the importance of environmental factors on the adaption of the pathogen [9]. *Xylella fastidiosa* colonizes plants living in environments with history of Cu-based antimicrobial compounds application, like those used in vineyards and orchards [10,11,12]. Although *X. fastidiosa*, which lives in xylem vessels of host plants, is not directly in contact with these Cu-based antimicrobials, external treatment with Cu antimicrobials can result in increased Cu levels in the soil and therefore in the xylem. Moreover, *X. fastidiosa* virulence is influenced by the level of Cu inside the host plant [12].

Research in *Xanthomonas* spp. isolated from 12 different countries showed that Cu resistant strains were not distributed evenly in these locations [13]. Cu resistance developed in some strains of *Xanthomonas* spp. in relation to frequent sprays of Cu-based bactericides as well as horizontal transfer of Cu resistance genes [13,14]. A An important difference between *Xanthomonas* spp. and *X. fastidiosa* is that the homologues of Cu-related genes (*copA*, *copB*, *copL,* and *cutC*) are only found in the chromosome of *X. fastidiosa* and not in plasmids [15]. Since these genes are closely related to Cu homeostasis genes in *Xanthomonas*, these were classified as Cu homeostasis genes rather than Cu resistance genes [15,16]. Previous results from our group showed that these Cu homeostasis genes in *X. fastidiosa* played a key role in withstanding Cu stress under different conditions and influenced *in planta* virulence [12,15].

Based on these preliminary observations, we sought to determine whether there is an association between Cu homeostasis ability of *X. fastidiosa* isolates and their phylogenetic relationships, subspecies classification, geographical location, or host species. In this study, this question was investigated by assessing the Cu minimum inhibitory concentration (MIC) of *X. fastidiosa* strains and discussing the relationships between phylogeny of four Cu-related genes as well as geographical locations and host species of strains. Lastly, these relationships were studied in a small group of *X. fastidiosa* strains isolated form Georgia’s vineyards.

## 2. Results and Discussion

### 2.1. Cu Homeostasis Ability (Cu MIC) of X. fastidiosa Strains

**Figure 1:**
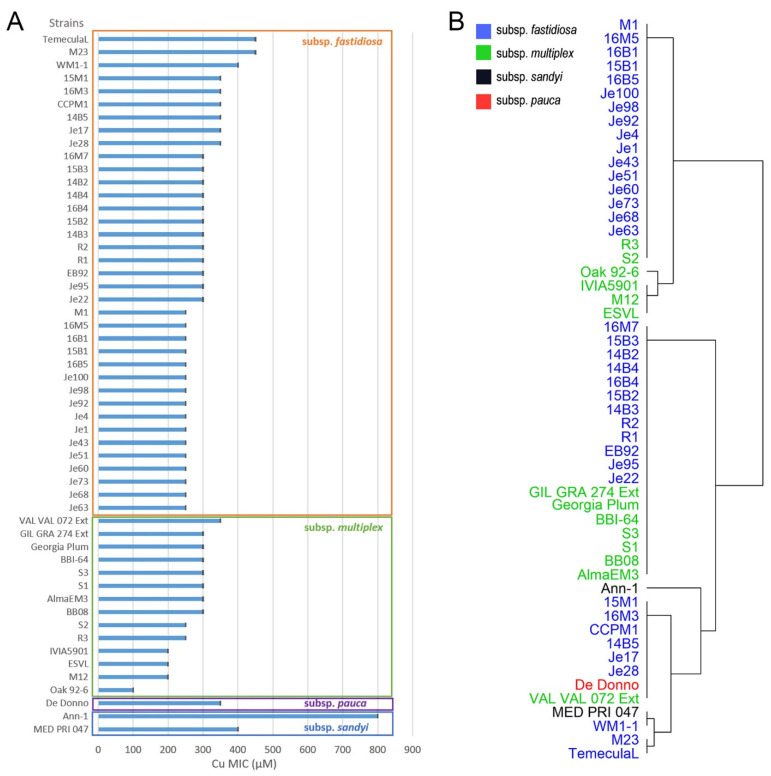
Cu MIC of *X. fastidiosa* strains. (**A**) Cu MIC values of strains, (**B**) Dendrogram of strains. Cu MIC assessment of *X. fastidiosa* strains were carried out in 96-well plates with PD2 media amended with different concentrations of CuSO_4_. The initial OD_600nm_ of bacteria culture was set as 0.01. PD2 media without any bacteria was considered as blank. Growth of bacteria was determined by total growth as a summation of biofilm growth and planktonic growth, which was measured at OD_600nm_. The minimal Cu concentration that significantly (*p* < 0.05) inhibited 90% bacterial total growth compared with the growth under PD2 media without Cu amendment was selected as Cu MIC. Three independent experiments were carried in this study with a total of *n* = 9 replications. Data represents means and standard error of the mean.

The in vitro MIC of CuSO_4_ of *X. fastidiosa* was used as numerical estimator of Cu tolerance among *X. fastidiosa* strains. In total, 54 strains of *X. fastidiosa* from 4 different subspecies were used in this study. To compare strains from different subspecies, these strains were grown and treated under the same conditions with increments of 150 to 900 µM CuSO_4_. In general, the range of Cu MIC were between 250 to 450 µM for strains of *X. fastidiosa* subsp. *fastidiosa*, and between 100 to 350 µM for strains of *X. fastidiosa* subsp. *multiplex* (Figure 1A). Due to quarantine restrictions in the US, only one strain from *X. fastidiosa* subsp. *pauca* was available for our studies. The Cu MIC of *X. fastidiosa* subsp. *pauca* strain De Donno was 350 µM. The Cu MIC of *X. fastidiosa* subsp. *sandyi* strain MED PRI 047 and Ann-1 were 400 and 800 µM, respectively. Among subspecies, differences among strains were evident (Figure 1A). For instance, in *X. fastidiosa* subsp. *fastidiosa* strains M23 and TemeculaL were significantly higher than other strains from the same subspecies. Strain Oak 92-6 with Cu MIC of 100 µM is significantly lower than other stains from *X. fastidiosa* subsp. *multiplex*. Cu MIC of strain Ann-1 from *X. fastidiosa* subsp. *sandyi* was higher compared with the other strains. The Cu MIC values of *X. fastidiosa* strains were found to be clustered in several groups, as shown in the dendrogram (Figure 1B). In general, strains could be divided into three big clusters related to low, medium, and high Cu MIC. The strains in low and medium Cu MIC cluster were all from subsp. *fastidiosa* and subsp. *multiplex*. Notably, these clusters were composed of strains from different subspecies, and no association between subspecies and MIC was detected here.

### 2.2. Phylogenetical Relationships of Cu-Related Genes copA, copB, copL, and cutC Follows X. fastidiosa Subspecies Classification and Was Not Related with Cu MIC

**Figure 2:**
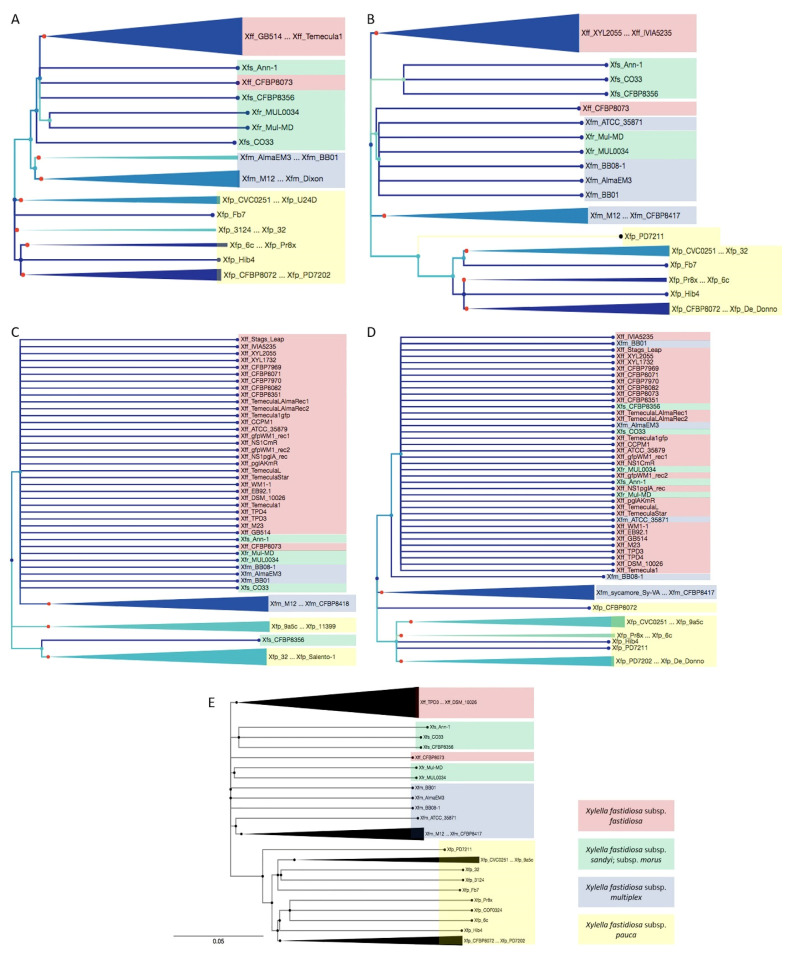
Phylogenetic trees of Cu-related genes in *X. fastidiosa*. Phylogenetic trees of *copA* (**A**); *copB* (**B**); *copL* (**C**); *cutC* (**D**), and *copLAB* (**E**) genes. Maximum-likelihood phylogenetic trees were built using MEGA 7.0. Branches below 70% of bootstraps values were collapsed. The strains were named according to subspecies classification as follows: Xff: *X. fastidiosa* subsp. *fastidiosa*; Xfm: *X. fastidiosa* subsp. *multiplex*; Xfs: *X. fastidiosa* subsp. *sandyi*; Xfr: *X. fastidiosa* subsp. *morus*; Xfp: *X. fastidiosa* subsp. *pauca*. For representation purposes, branches were collapsed when the strains belong to same subspecies in a cluster, and genes were color-coded according to the subspecies classification of their strain.

To understand the difference in Cu MIC among *X. fastidiosa* strains, we completed a phylogenetic analysis of the Cu-related genes (*copA*, *copB*, *copL,* and *cutC*) to assess differences at the gene sequence level among *X. fastidiosa* strains. The Cu-related genes (*copA*, *copB*, *copL* and *cutC*) were identified from genomes of 74 *X. fastidiosa* strains using nucleotide BLAST based on the annotated genes from type strain Temecula1. Phylogenetic trees were made by using the maximum-likelihood method [17] (Figure 2).

Overall, high DNA sequence identity of the Cu-related genes was identified among strains, with above 95% for *copA*, *copL,* and *cutC*; and above 90% for *copB*. This result was similar to that observed in *Xanthomonas* spp. with >90% DNA sequence identity in Cu resistance genes *copL*, *copA,* and *copB* [13]. Phylogenetic analysis highlighted the slight differences among the sequences of these genes among strains and separated them in different groups. The sequences for *copL* were highly conserved among the strains with the majority of sequence present in a polytomy. This conservation may suggest a tight homeostatic regulation of Cu across populations of *X. fastidiosa*. A previous study of homologues of *copL* in *Xanthomonas* spp., a gene related with transcriptional and translational regulation of *copAB* operon, also highlighted the expected conservation of *copL* in these related pathogens [18]. The sequences for *cutC* were also highly conserved among the isolates. *copA* and *copB* were less conserved compared with *copL* and *cutC*. The phylogeny analysis was also carried with *copLAB* genes concatenated together (Figure 2), which showed similar tree topology as phylogenetic trees for *copA* and *copB* genes. The phylogenetical relationships of these genes followed the *X. fastidiosa* subspecies classification with few exceptions.

To be more specific, similar grouping and cluster patterns of phylogenetic trees were found between *copA* and *copB*, *copL* and *cutC*. In *copL* and *cutC* phylogenetic trees, the strains from *X. fastidiosa* subsp. *sandyi* (Xfs), *X. fastidiosa* subsp. *multiplex* (Xfm), and *X. fastidiosa* subsp. *morus* (Xfr) were mixed with *X. fastidiosa* subsp. *fastidiosa* (Xff). However, the genes from these strains were closely related with Xff but separated in different clusters in *copA* and *copB* phylogenetic trees. Interestingly, an exception was the *copL* gene of Xfs strain CFBP8356 that was closely related to *X. fastidiosa* subsp. *pauca* (Xfp) instead of the other Xfs strains (Figure 2C); the other three genes analyzed of this strain were grouped with Xfs. However, when comparing Cu MIC values of the tested strains and their phylogeny here, it was noticed that strains within the same group did not follow the same clustering based on Cu MIC value (Figure 1B). For instance, strains TemeculaL, CCPM1, and EB92-1 were in the same group in all the phylogenetic trees, while, they had Cu MIC at 450, 400, and 350, respectively, which were not in the same cluster (Figure 1B). The Cu MIC value of Xfs strain CFBP8356, which was noticed for its special phylogenetic grouping, was not tested in this study as the strain was not available. Studies in *Xanthomonas* spp. showed that the phylogenetic analysis of Cu-resistant strains revealed a geographic-based grouping pattern [13]. Although *X. fastidiosa* is genetically closely related with *Xanthomonas* spp., the differentials of living environment and virulence characteristics may be responsible for the lack of unique phylogenetic groupings based on their Cu-related genes. The differences in Cu MIC values of *X. fastidiosa* strains could not be explained by the Cu-related genes phylogeny.

The sequence variation alone could not explain the difference in Cu MIC so we analyzed selection pressure and codon adaption index (CAI) assessment of the Cu-related genes. Datamonkey.org was used in this study to assess the strength of selection pressure on *copA* and *copB*, *copL,* and *cutC* genes. The rate of non-synonymous substitution (dN) and random synonymous substitution (dS) were assessed in each gene. Positive selection (dN/dS > 1) increases the diversity and fitness of genes in response to environmental pressure, while negative selection (dN/dS < 1) removes and purifies these changes [19]. The results of the analysis showed that when *p*-value threshold was set at 0.05, *copA* had one codon site (site 429) under significant positive selection, and 14 sites under significant negative selection. There were 10, 2, and 3 sites under significant negative selection in *copB* gene, *copL* gene and *cutC* gene, respectively, while none of them had any significant positive selected site (Table 1). Analysis results showed that the four genes were under negative selection, as there were more random synonymous substitutions (dS) than non-synonymous substitutions (dN) [19,20]. Negative selection can happen more often in functionally important genes to avoid changing the essential functions by non-synonymous substitution [21]. Therefore, the *copA* and *copB*, *copL,* and *cutC* genes that had lower dN/dS ratios and are undergoing purifying selection are believed to serve important roles in *X. fastidiosa*. CAI assessment is positively associated to gene expression level and therefore, we analyzed the four Cu-related genes by comparing their codon usage frequency with codon usage frequency of the reference gene [22,23,24]. *Tu* gene, which is highly expressed across many bacteria and unicellular eukaryotes, was used as the reference gene in this study [23,24]. Gene with CAI value higher than that of *Tu* gene was considered as being highly expressed [23]. CAI assessment results (Figure S1) of *copA* and *copB*, *copL,* and *cutC* genes showed that *copA* gene displayed slightly but significantly higher CAI value compared with *Tu* gene, which was considered a highly expressed gene. However, the other three genes had significantly lower CAI values compared with the reference gene. The higher CAI value of *copA* than the other three genes were consistent with the results of selection pressure analysis of the four genes, in which *copA* has more synonymous substitutions than the other three genes. The results of selection pressure analysis and CAI assessment indicated that *copA* and *copB*, *copL,* and *cutC* genes were functionally important for *X. fastidiosa*. However, there was no evidence indicating that the differences in gene level was associated with the difference of Cu homeostasis ability among different subspecies.

### 2.3. Cu MIC of X. fastidiosa Strains Is Not Significantly Associated with Subspecies, Host, or Location

**Figure 3:**
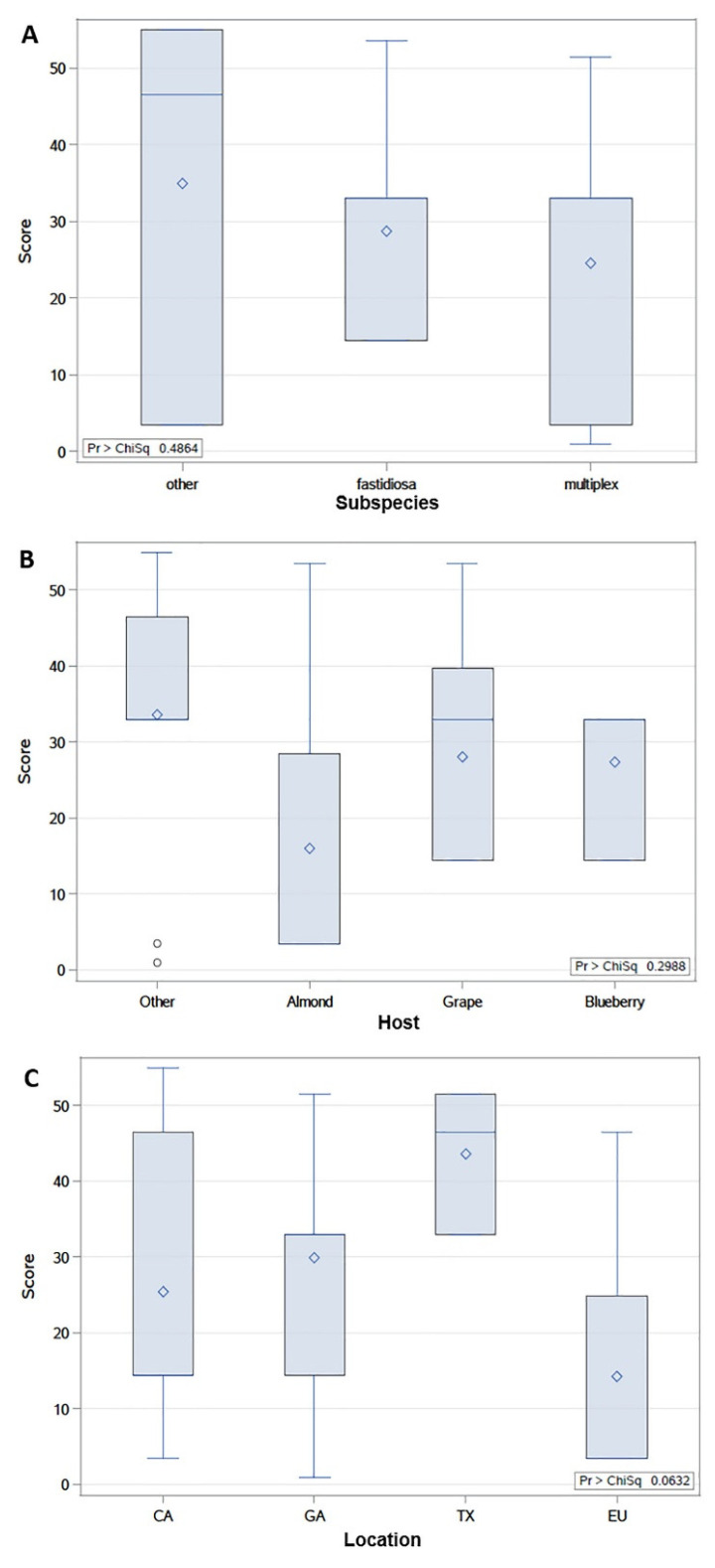
Rank score of Wilcoxon test between Cu MIC and strains classification and origin. Rank scores of Wilcoxon test were used to indicate if the Cu MIC from each group were differently distributed from each other. Strains were grouped based on their (**A**) subspecies: fastidiosa (*X. fastidiosa* subsp. *fastidiosa*), multiplex (*X. fastidiosa* subsp. *multiplex*), and other (*X. fastidiosa* subsp. *pauca* and *X. fastidiosa* subsp. *sandyi)*; (**B**) host: grape, blueberry, almond, and other (olive, oleander, elderberry, plum, giant ragweed, sunflower and oak); (**C**) location: CA (California), GA (Georgia and Florida), TX (Texas) and EU (Spain and Italy). *p* value (Pr > ChiSq) < 0.05 was considered as a threshold for statistical significance carried by the Kruskal-Wallis test.

Although differences among Cu MIC in *X. fastidiosa* strains were not associated with phylogeny of Cu-related genes, their relationship with subspecies, host type, and geographical location were examined to understand if there were associations among them. Cu MIC values of *X. fastidiosa* strains were grouped based on their classification and origin (subspecies, host, or location). Strains with similar subspecies, host, or geographical origin were combined in the same group whenever few strains were available with the same characteristics. The differences of Cu MIC values distributions among each group, measured by scores from Wilcoxon tests, were used in this study to indicate if the grouping-based characteristic was associated with the Cu MIC value of *X. fastidiosa* strains. The results showed that Cu MIC in *X. fastidiosa* strains was not markedly different amongst subspecies, the mean rank scores from the test of the Cu MIC data in others (subsp. *pauca* and subsp. *sandyi*), subsp. *fastidiosa* and subsp. *multiplex* were 35, 28, and 24, respectively (Figure 3A). Mean rank scores were different in subspecies groups; however, there was no statistical difference (*p* = 0.48) (Figure 3A). Therefore, the distribution of Cu MIC in each group of subspecies of *X. fastidiosa* was not significantly differentiated from each other, which indicated that the subspecies classification of *X. fastidiosa* strains was not significantly associated with Cu MIC. Furthermore, the relationship between Cu MIC and *X. fastidiosa* strains features, including host and location, were studied (strains information is listed in Table S1). Based on the results of Wilcoxon test, similar conclusions could be drawn; different mean rank scores were found among the host and location groups (Figure 3B,C). However, in both cases, *p*-values were higher than 0.05; therefore, no significant differences were found. Host or location of *X. fastidiosa* strains was not significantly associated with Cu MIC.

Based on the results of the 54 strains tested in this study, no significant association was found between their Cu MIC and their features, including subspecies, host, or location. However, the limitation in this study was that the sample size in each group was uneven, due to the limited availability of *X. fastidiosa* strains from different groups. It is still possible that some relationships exist between the Cu homeostasis and isolates characteristics that could be revealed with a more comprehensive study. Previous research in *Xanthomonas* spp. indicated a possible location-based distribution pattern of Cu resistance strains, which might be a consequence of the application of Cu-based compounds and horizontal transfer of Cu resistance genes [13,14]. In addition, Cu contained in agricultural soil was believed to be positively associated with environmental antibiotic resistances [25,26]. Environmental Cu accumulation also could trigger the evolution of Cu resistance genes in pathogens [27]. Therefore, the soil Cu content in a region could be a dominant feature that influences Cu homeostasis of *X. fastidiosa* isolated from that region as well as the agriculture practices in use in different settings. However, lacking such information at the vineyard/farm level could be an obstacle to reveal patterns of Cu homeostasis among different *X. fastidiosa* strains.

### 2.4. X. fastidiosa Isolates from Georgia’s Vineyards Have Similar Responses of Cu Accumulation and Biofilm Formation under Cu Treatment

**Figure 4:**
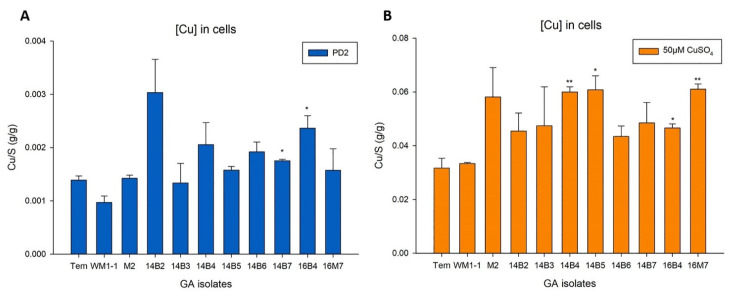
Cu accumulation in the isolates from Georgia’s vineyards. Cu accumulation in cells when grown in (**A**) PD2 media and (**B**) PD2 media amended with 50 µM CuSO_4_. Cu content was measured by ICP-OES. Isolates were grown in test tubes for 7 days with PD2 media or PD2 amended with 50 µM CuSO_4_. Mean values were shown in graph, and error bars represented standard error of the mean (*n* = 6). Different Y scales were used in this figure since values in A were between 0–0.004; while values in B were between 0–0.08. Data used in the graph corresponded to one representative experiment, and three independent experiments performed under the same conditions showed similar tendencies. * and ** indicated significant differences (*p* < 0.05 and *p* < 0.01, respectively) compared with control strain TemeculaL (“Tem”) according to the two-tailed Student’s *t*-test.

Although the above results did not indicate a significant association between Cu homeostasis ability and strains features, a further investigation on a smaller group of *X. fastidiosa* strains were carried out. *X. fastidiosa* isolated from grapevines grown in vineyards in the same county in Georgia (GA, USA) were selected to be studied. Copper levels in the isolates with or without Cu-amended treatments was assessed. *X. fastidiosa* type strain TemeculaL (isolated from grapevines in California) was used as a control. The isolates and the control strain were grown in Pierce’s Disease 2 (PD2) media, and PD2 media amended with 50 µM CuSO_4_. Cu accumulation in cells of each isolate was measured by Inductively Coupled Plasma with Optical Emission Spectroscopy (ICP-OES). Results (Figure 4A) indicated that most of the isolates from GA had slightly higher Cu accumulation in cells than TemeculaL, with two isolates (14B7 and 16B4) showing statistically significant differences (*p* < 0.05), compared with control strain when grown in PD2 media. The difference between the isolates and control strain was more pronounced under Cu-amended conditions. Cu accumulation in cells of the isolates were higher than that of control, with four isolates significantly different (*p* < 0.05) (Figure 4B). Overall, the isolates from Georgia’s vineyards accumulated more Cu than control strain both under normal condition (PD2 media) and Cu-amended condition (PD2 amended with 50 µM CuSO_4_). However, there were some exceptions; for example, WM1-1 had lower or similar Cu content in cells as control strain under the same conditions. WM1-1 [28] was isolated from the same region as the other GA isolates used here, but a few years earlier. Perhaps cultivation under laboratory conditions for several years could be a factor for leading this difference. Another possible reason could be the difference of antimicrobials and management methods used in vineyards, as WM1-1 was from Wolf Mountain Vineyard, and the others were from Blackstock Vineyard and Montaluce Vineyard.

When considering the Cu MIC results of the GA isolates (Figure 1), most of the isolates had Cu MIC ranging from 250 to 300 µM. Only 3 isolates with Cu MIC value were equal to 350 µM and 1 isolate Cu MIC was 400 µM. However, the Cu MIC of the control strain was 450 µM. Generally, the isolates from Georgia’s vineyards were more sensitive to Cu than control strain TemeculaL. The results of Cu accumulation and Cu MIC are consistent with each other, since more Cu accumulation in cells lead to a build-up of toxicity, and lower Cu MIC for the isolates. Moreover, TemeculaL comes from vineyards with longer history in CA, while vineyards in GA are newer. It is worth noticing that TemeculaL has been used in culture under laboratory conditions for many years, while strains from GA are not. Researchers have previously shown that Cu content in vine and soil are closely related with the age of vineyards [29,30]. It is possible that GA isolates were less exposed to Cu compared with TemeculaL, which makes them more sensitive to Cu. There are clear genomic difference and grouping between whole genomes of CA and GA isolates [31]. However, there was no significant differences between CA and GA isolates in the four Cu-related genes discussed in this study (Figure S2). Even though the CA and GA isolates had slightly difference in the phenotype of Cu MIC, it was not clearly associated with *copA*, *copB*, *copL,* and *cutC* genes phylogeny.

**Figure 5:**
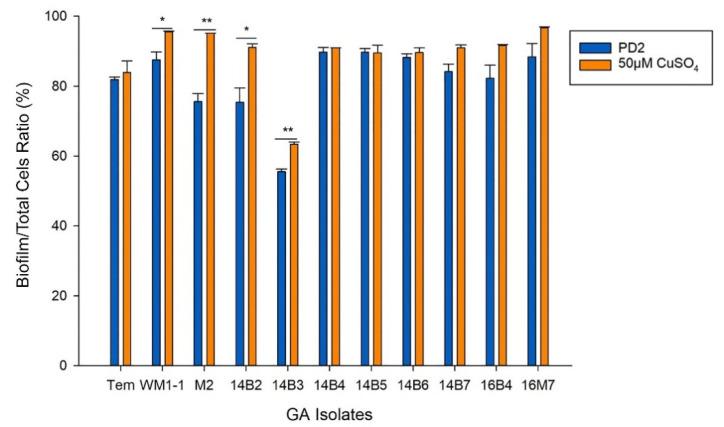
Biofilm/total cells ratios of the isolates from Georgia’s vineyards. Biofilm/total cells ratio was measured by sulfur (S) concentration in biofilm cells divided by S concentration in total cells, as cellular S content is linearly associated with cell number. Bacteria were grown under PD2 media or PD2 amended with 50 µM CuSO_4_. After 7-day incubation in PD2 media or PD2 media amended with 50 μM CuSO_4_, the biofilm cells and planktonic cells were carefully separated with pipetting and collected by centrifugation. The data used in the graph corresponded to means and standard errors of the mean from one representative experiment, and three independent experiments performed under the same conditions showed similar tendencies. * and ** above the black line indicated significant differences (*p* < 0.05 and *p* < 0.01, respectively) of biofilm/total cells ration when grown in PD2 media and PD2 media amended with 50 μM CuSO_4_, according to the two-tailed Student’s *t*-test.

Biofilm formation is one of the key virulence factors of *X. fastidiosa* [32]. Therefore, biofilm formation of *X. fastidiosa* isolates from Georgia’s vineyards under Cu treatment was investigated. Biofilm formation of the isolates under PD2 and PD2 amended with 50 µM CuSO_4_ conditions were analyzed and compared with control strain TemeculaL (Figure 5). Most of the isolates had higher biofilm/total cells ratios when grown in PD2 media, except for isolates M2, 14B2, and 14B3, which had lower biofilm/total cells ratio. When grown in Cu-amended media, increase of biofilm/total cells ratios was observed in all the isolates, with 4 isolates showing significant increase (*p* < 0.05). 14B3 showed significantly lower biofilm/total cells ratio compared with other isolates and the control strain, even under the Cu-amended condition. However, isolate 14B3 was not significantly different from other strains in Cu MIC assessment or in the assessment of Cu accumulation in cells. The reason for its low biofilm/total cells ratio in all the tested conditions may not be related to Cu homeostasis, as it has the same response to Cu amendments as the other isolates. The results showed that Cu was able to not only induce biofilm cell growth (as shown in previous research [33]), but more importantly also increase the proportions of biofilm cells in comparison to total cells, which potentially increased *X. fastidiosa in planta* fitness and virulence [32,34]. Meanwhile, the overall increase of biofilm/total cells ratio may be related to the metabolic consequences of increased Cu accumulation in cells under the Cu-amended condition.

## 3. Materials and Methods

### 3.1. Cu MIC Measurements

Cu MIC measurements, considered here as the Cu concentration inhibiting 90% of growth or MIC_90_, were performed following protocols described in previous publications [35,36], with some modifications. Bacterial isolates and strains were streaked onto Periwinkle Wilt (PW) agar plates from -80 ℃ frozen glycerol (20%) stocks. After 5–7 days of first streaking, bacteria were re-streaked onto a new PW agar plate for another 5–7 days. After the second streaking, these bacterial cells were ready to use for later experiments. Bacterial cells were scraped from PW agar plates and suspended into PD2 liquid media. The optical density (OD_600nm_) of bacterial suspensions were adjusted to 0.2 for later use. 190 µL PD2 media or PD2 media amended with different concentrations of CuSO_4_ (0 µM, 150 µM, 200 µM, 250 µM, 300 µM, 350 µM, 400 µM, 450 µM, 500 µM, 600 µM, 700 µM, 800 µM, 900 µM) and 10 µL bacterial suspensions (OD_600nm_ = 0.2) were added to each well of 96-well plate. The final concentration of bacteria in each well was OD_600nm_ = 0.01. Bacteria cultured in 96-wells plates were incubated with 140 rpm shaking condition at 28 ℃. After 7-days incubation, 150 µl of bacterial culture was transferred to a new 96-wells plate, and OD_600nm_ was measured for planktonic growth calculations. For biofilm growth measurements, original plates were rinsed with Milli-Q water twice to remove planktonic cells. Then, 230 μL of 0.1% crystal violet was added to each well and incubated at room temperature for 20 min with frequent shaking. After that, crystal violet was removed carefully from each well with pipettes. Plates were rinsed with Milli-Q water 2 to 3 times. Finally, each well was filled with 230 μL 95% ethanol and incubated for 5 to 10 min. OD_600nm_ values were measured to calculate biofilm formation. All the procedures of adding and removing liquid were performed under gentle and carefully pipetting, to minimize the loss of biofilm cells. The minimal Cu concentration that significantly (*p* < 0.05) decreased 90% of total bacterial growth (total growth = planktonic growth + biofilm growth) was selected as Cu MIC. Dendrogram of Cu MIC showing the results of 54 *X. fastidiosa* strains after a hierarchical clustering analysis of Cu MIC values was carried with R 3.6.2 (Rstudio 1.1.442, Boston, MA, USA) using between-group linkage via Ward’s hierarchical clustering.

### 3.2. Phylogenetic Analysis

Phylogenetic trees were constructed using Molecular Evolutionary Genetics Analysis version 7.0 (MEGA7) software (Kumar, Stecher, and Tamura, PSU, PA, USA). Whole genomes of each isolate and strain were obtained from sequence results of our lab or Almeida’s lab [31], or from the National Center for Biotechnology Information (NCBI) database (Table S2). Then, the target gene sequences for each isolate were selected by blasting whole genomes with corresponding genes from Temecula1 by using Basic Local Alignment Search Tool for Nucleotides (BLASTN). After that, partial nucleotide sequences of Cu-related gene *copL* (PD0099), *copA* (PD0100), *copB* (PD0101), and *cutC* (PD0586) were aligned by default settings of MUSCLE in MEGA7. The alignments were assembled into a maximum-likelihood tree with 1000 bootstraps. The bootstrap values, as percentage out of 1000 replicates that the associated strains were clustered together in the bootstrap test, are shown next to the branches (Figure S3). Branches with bootstrap values below 70% were collapsed. Phylogenetic trees were further adjusted in Phylo.io (www.Phylo.io accessed 20 June 2020) to make it easier to view [17]. Branches were collapsed when all the strains in a cluster belong to the same subspecies.

### 3.3. Selective Pressure Analysis and Expression Prediction

To assess if *copL*, *copA*, *copB,* and *cutC* genes were under selective pressure, analysis were carried in Datamonkey adaptive evolution server (www.Datamonkey.org accessed on 19 August 2020) [20]. Alignment of each gene was obtained by using the default settings of MUSCLE in MEGA7. Stop codons in the alignment of each gene were removed as required by the instructions of the software [19]. Alignments were uploaded to the website as meg format. Selective pressure analysis of the genes was carried by the fixed effects likelihood (FEL) method with default settings. Positive (diversifying) selection for each codon was considered as non-synonymous substitution (dN) > random synonymous substitution (dS) (dN/dS > 1); while negative (purifying) selection was considered when dN < dS (dN/dS < 1), with *p* value < 0.05 [19]. Gene expression was predicted by CAI (codon adaption index) calculation, carried by CAIcal (www.ppuigbo.me/programs/CAIcal accessed on 15 September 2020) [23,37,38]. The *Tu* gene, an elongation factor gene that is highly expressed in many organisms, was used as the reference gene [24]. CAI value higher than the *Tu* gene was considered as a high gene expression level.

### 3.4. Cu Accumulation in Cells

Cu content in bacterial cells after Cu-amended treatments were measured by ICP-OES (Perkin Elmer 7100 DV, Waltham, MA, USA) as previously described [12,33], with some modifications. Bacteria were grown in 5 mL PD2 media or PD2 media amended with 50 μM CuSO_4_, with initial OD_600nm_ equal to 0.01. Bacterial cells were collected after 7-day incubation and washed twice in Milli-Q water to remove media. In each tube, 100 μL of mineral-free concentrated nitric acid (OPTIMA, Fisher Scientific, Waltham, MA, USA) were added in each sample for digestion. The digestion was processed under 100 °C heat treatment for one hour. Later, samples were diluted with 200 μL Milli-Q water. The analysis was carried by ICP-OES with simultaneous measurement of Ca, Fe, Cu, Zn, Mn, S, Mg, K, Na, and P. Concentrations of mineral elements in each sample were determined by comparing emission intensities to certified standards curves (SPEXCertiprep, Metuchen, NJ, USA), which was confirmed by the reanalysis of standard solutions diluted in a matrix equivalent to the sample [12,33]. Two repetitions of individual readings (each individual reading is average of two intensity measurements) showed less than 5% variation.

### 3.5. Biofilm/Total Cells Ratio

Biofilm/total cells ratio was calculated using the sulfur (S) concentration obtained by ICP-OES as follows: S concentration in biofilm cells/S concentration total cells. Bacteria were prepared and cultured as described above for measurements of Cu accumulation. After 7-day incubation under PD2 media or PD2 media amended with 50 μM CuSO_4_, biofilm and planktonic cells were carefully separated with pipetting and collected by centrifugation. Cells were washed with Milli-Q water to remove media and measured by ICP-OES under the same conditions. The S content in cells was linearly associated with the cell number, and was used as an indicator of cell numbers [33]. Therefore, here biofilm/total cells ratio was represented by S content in biofilm cells/total cells.

### 3.6. Association Tests

To understand the relationship between Cu MIC values of *X. fastidiosa* strains (quantitative variables) and strains characteristics (qualitative variables), including subspecies, location and host, commonly used correlation tests were not applied since they require quantitative variables. In here, the association between Cu MIC values of *X. fastidiosa* strains and strains characteristics were performed by comparing the Cu MIC data distributions in each variable groups (comparisons happened when only one characteristic variable at a time). Scores from Wilcoxon test indicated the degree of the difference of Cu MIC data distribution among each variable groups. If scores were significantly different between groups, it means the grouping-based characteristic could be the factor that influences the distribution of Cu MIC values in the groups, which suggests the characteristic was associated with Cu MIC values of strains. *p* value (Pr > ChiSq) < 0.05 was considered as a threshold for statistical significance by Kruskal-Wallis test (nonparametric ANOVA). These analyses were carried by SAS 9.4 (SAS Institute, Inc., Cary, NC, USA). The Cu MIC value of strains were grouped based on their characteristics, and the groups with small samples size were combined. Strains were grouped based on subspecies: fastidiosa (*X. fastidiosa* subsp. *fastidiosa*), multiplex (*X. fastidiosa* subsp. *multiplex*), and other (*X. fastidiosa* subsp. *pauca* and *X. fastidiosa* subsp. *sandyi)*; based on host: grape, blueberry, almond, and other (olive, oleander, elderberry, plum, giant ragweed, sunflower and oak); based on location: CA (California), GA (Georgia and Florida), TX (Texas) and EU (Spain and Italy).

## 4. Conclusions

In this study, Cu homeostasis in vitro (MIC) of a collection of 54 *X. fastidiosa* strains was found to not be related with either the strain geographic or plant host origin of isolation, or their phylogenetic relationships. Although no clear tendencies were found among these strains, the study of Cu-related characteristics on a small sub-group of *X. fastidiosa* isolates from Georgia indicated a possible connection between Cu homeostasis ability and their Cu-related phenotypes. The *X. fastidiosa* isolates, obtained from the similar host (grapevine varieties) and the similar locations (vineyards in Dahlonega, Georgia), mostly responded similarly to Cu amendments. These isolates had higher Cu accumulation in cells and lower Cu MIC than the control strain (TemeculaL). Based on the preliminary data from this study, the variability in Cu homeostasis ability is not explained by either phylogeny, subspecies, location, or host of *X. fastidiosa* strains. Limited and uneven sample size of each feature group, due to limited availability of *X. fastidiosa* strains, might have been a problem to answer our questions. Further research with more strains could help us better understand the importance of Cu homeostasis ability to the *X. fastidiosa* strains from different geographic locations or host species. This will help understand the adaptation of *X. fastidiosa* to different environments and provide novel ideas for worldwide *X. fastidiosa* control and its disease management.

## Figures and Tables

**Table 1:** Summary of selection in the Cu-related genes.

Gene	# Positive Sites ^1^	# Negative Sites ^2^	# Total Sites ^3^
*copA*	1	14	611
*copB*	0	10	250
*copL*	0	2	126
*cutC*	0	3	246

^1^ Positive sites is when non-synonymous substitution (dN) is significantly (*p* < 0.05) higher than random synonymous substitution (dS) (dN/dS > 1). ^2^ Negative sites is when dN is significantly (*p* < 0.05) lower than dS (dN/dS < 1). ^3^ Total sites is the total codon sites (including non-significant sites) that are possibly under selection searched by Fixed Effects Likelihood method (FEL) method carried with Datamonkey.org.

## Data Availability

Data is contained within the article or supplementary material.

## Supplementary Materials

The following are available online at https://www.mdpi.com/article/10.3390/pathogens10040495/s1, Figure S1: Range of CAI of *copA*, *copB*, *copL*, and *cutC* genes, Figure S2: Phylogenetic trees of *copA*, *copB*, *copL*, and *cutC* genes from the strains used for Cu MIC assessment in vitro, Figure S3: Phylogenetic trees of *copA*, *copB*, *copL*, and *cutC* genes. Table S1: Information of *X. fastidiosa* isolates and strains for Cu MIC assessment, Table S2: Information of *X. fastidiosa* isolates and strains for phylogeny.
