# Supplementary material for: Phenotypic and Phylogenetic Characterization of Cu Homeostasis among Xylella fastidiosa Strains
Source: Pathogens. 2021 Apr 20;10(4):495. doi: 10.3390/pathogens10040495 (PMC8073393; doi:10.3390/pathogens10040495)
Supplement: Supplementary file 1 [file pathogens-10-00495-s001.zip › pathogens-1184409-supplementary/supp new/Figure S1.pdf]

Figure S1: Range of CAI of *copA*, *copB*, *copL* and *cutC* genes.

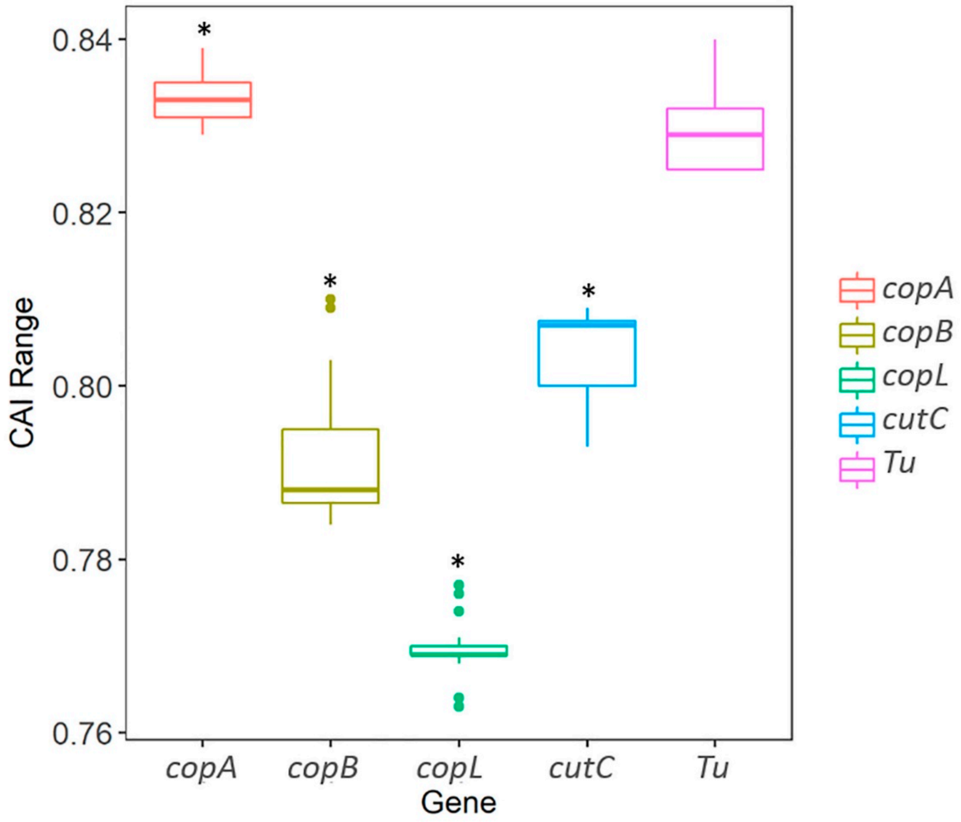

Range of CAI of *copA*, *copB*, *copL* and *cutC* genes assessed by CAIcal.

CAI (codon adaption index) calculations of *copA*, *copB*, *copL* and *cutC* genes were carried by CAIcal (www.ppuigbo.me/programs/CAIcal) as instructions [21-23]. *Tu* gene was used as reference gene. Symbol * above each box indicated significant difference (p<0.05) between each tested gene and *Tu* gene according to two-tailed Student's *t-test* (p <0.050) (Microsoft Excel® 2016 for Windows)
