# Supplementary material for: Phenotypic and Phylogenetic Characterization of Cu Homeostasis among Xylella fastidiosa Strains
Source: Pathogens. 2021 Apr 20;10(4):495. doi: 10.3390/pathogens10040495 (PMC8073393; doi:10.3390/pathogens10040495)
Supplement: Supplementary file 1 [file pathogens-10-00495-s001.zip › pathogens-1184409-supplementary/supp new/Figure S3.pdf]

Figure S3: Phylogenetic trees of *copA*, *copB*, *copL* and *cutC* genes.

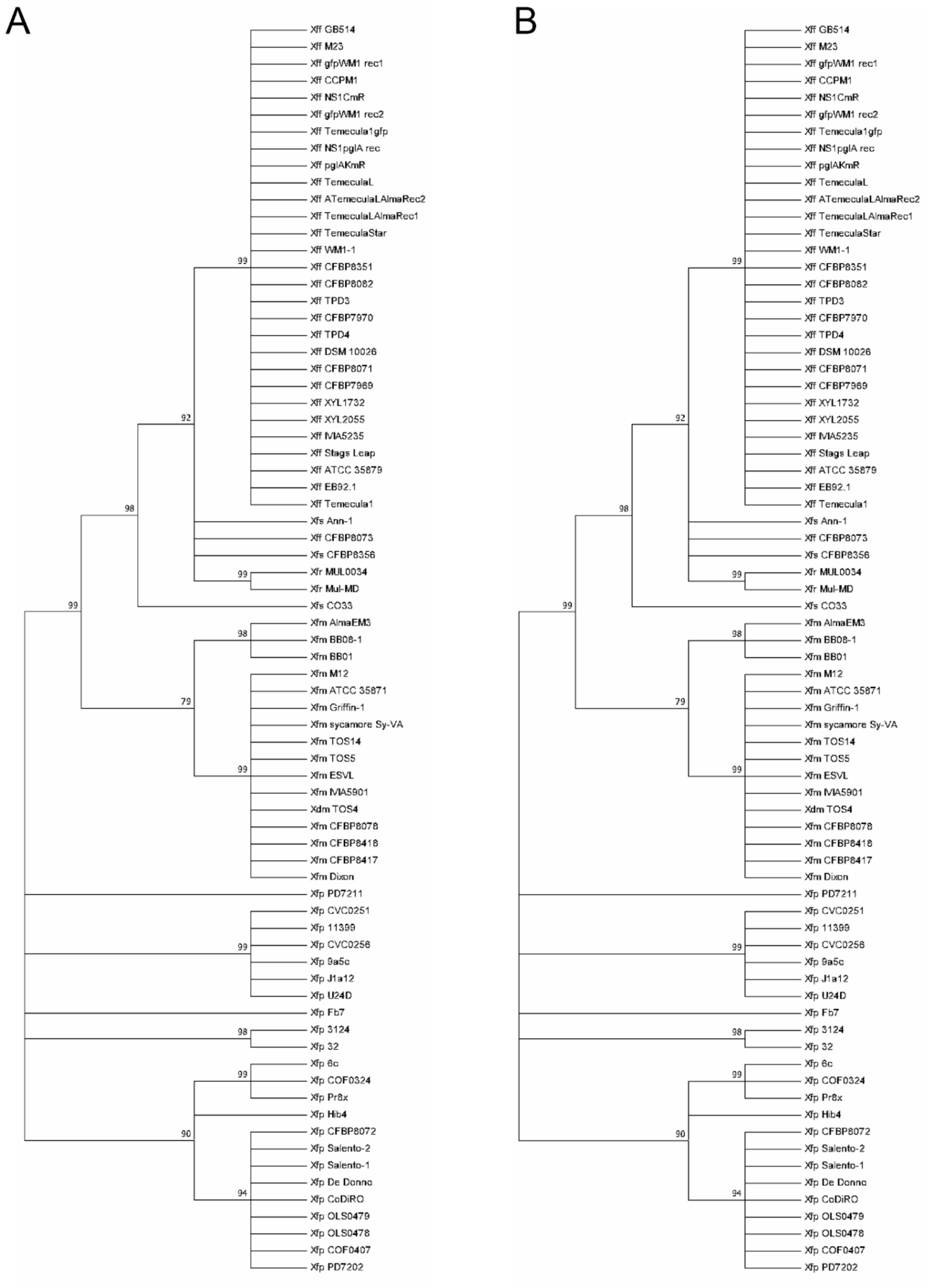

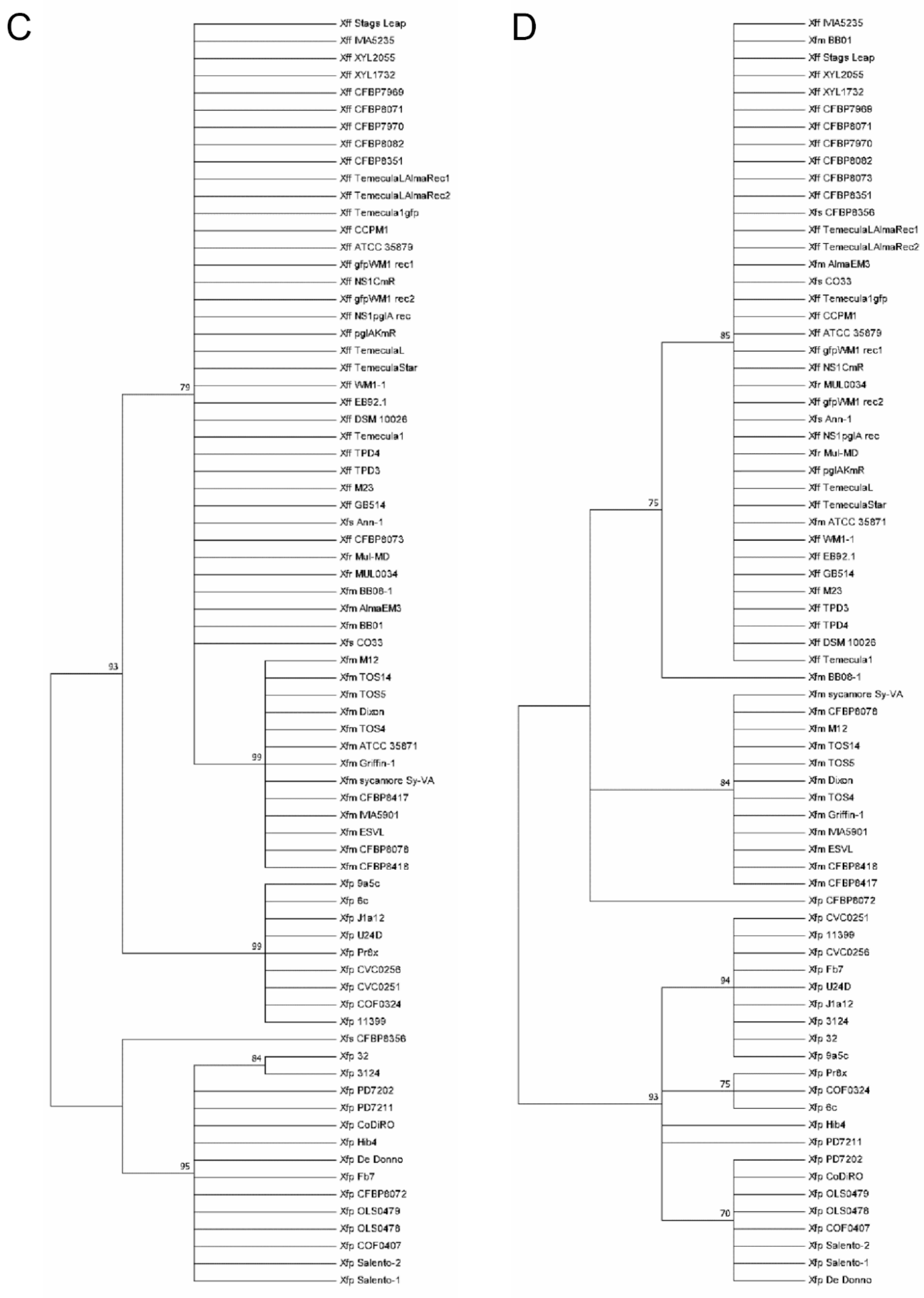

Phylogenetic trees of *copA*, *copB*, *copL* and *cutC* genes without collapsing strain names classified in the same cluster. Phylogenetic trees of (A) *copA*; (B) *copB*; (C) *copL* and (D) *cutC* genes. Maximum-likelihood phylogenetic trees were built using MEGA 7.0, Branches

below 70% of bootstraps value were collapsed. The strains were named according to subspecies classification as follows: Xff: *X. fastidiosa* subsp. *fastidiosa*; Xfm: *X. fastidiosa* subsp. *multiplex*; Xfs: *X. fastidiosa* subsp. *sandyi*; Xfr: *X. fastidiosa* subsp. *morus*; Xfp: *X. fastidiosa* subsp. *pauca*.
