# Supplementary material for: Phenotypic and Phylogenetic Characterization of Cu Homeostasis among Xylella fastidiosa Strains
Source: Pathogens. 2021 Apr 20;10(4):495. doi: 10.3390/pathogens10040495 (PMC8073393; doi:10.3390/pathogens10040495)
Supplement: Supplementary file 1 [file pathogens-10-00495-s001.zip › pathogens-1184409-supplementary/supp new/Table S1.pdf]

Supplemental Table S1: Information of *X. fastidiosa* isolates and strains for Cu MIC assessment.

Stains	Cu MIC (µM)	Subspecies	State	Location	Host	Host detail	Isolate Sources
**M23**	450	*fastidiosa*	CA	Kern	Almond	Rosaceae, Prunus dulcis	6
**Temecula L**	450	*fastidiosa*	CA	Temecula, Riverside	Grape	Vitaceae, *Vitis vinifera*	1
**WM1-1**	400	*fastidiosa*	GA	Wolf Mountain, Dahlonega	Grape	Mourvedre	1
**Je28**	350	*fastidiosa*	CA	Napa, Spring Mountain	Grape	Merlot	2
**Je17**	350	*fastidiosa*	CA	Sonoma, Newsome	Grape	Malbec	2
**14B5**	350	*fastidiosa*	GA	Blackstock, Dahlonega	Grape	Viognier	1
**CCPM1**	350	*fastidiosa*	GA	Cavender Creek, Dahlonega	Grape	Petite Manseng	1
**16M3**	350	*fastidiosa*	GA	Montaluce, Dahlonega	Grape	Merlot	1
**15M1**	350	*fastidiosa*	GA	Montaluce, Dahlonega	Grape	Montaluce	1
**Je22**	300	*fastidiosa*	CA	Napa, Spring Mountain	Grape	Merlot	2
**Je95**	300	*fastidiosa*	CA	Sonoma, Rudd	Grape	Chardonnay	2
**EB92-1**	300	*fastidiosa*	FL	Leesburg, Lake	Elderberry	Sambucus canadensis	4
**R1**	300	*fastidiosa*	GA	Bacon, Blake Williams Farm	Blueberry	Rebel	10
**R2**	300	*fastidiosa*	GA	Bacon, Blake Williams Farm	Blueberry	Rebel	10
**14B3**	300	*fastidiosa*	GA	Blackstock, Dahlonega	Grape	Cab Sauvignon	1
**15B2**	300	*fastidiosa*	GA	Blackstock, Dahlonega	Grape	Chardonnay	1
**16B4**	300	*fastidiosa*	GA	Blackstock, Dahlonega	Grape	Chardonnay	1
**14B4**	300	*fastidiosa*	GA	Blackstock, Dahlonega	Grape	Chardonnay	1
**14B2**	300	*fastidiosa*	GA	Blackstock, Dahlonega	Grape	Mourvedre	1
**15B3**	300	*fastidiosa*	GA	Blackstock, Dahlonega	Grape	Viognier	1

**16M7**	300	*fastidiosa*	GA	Montaluce, Dahlonega	Grape	Vidal	1
**Je63**	250	*fastidiosa*	CA	Bakersfield, Flames	Grape	Flames	2
**Je68**	250	*fastidiosa*	CA	Bakersfield, Flames	Grape	Flames	2
**Je73**	250	*fastidiosa*	CA	Bakersfield, Red Globe	Grape	Red Globe	2
**Je60**	250	*fastidiosa*	CA	Bakersfield, Scarlet Royal	Grape	Scarlet Royal	2
**Je51**	250	*fastidiosa*	CA	Napa, Silverado	Grape	Merlot	2
**Je43**	250	*fastidiosa*	CA	Napa, Yount Mill	Grape	Cabernet Sauvignon	2
**Je1**	250	*fastidiosa*	CA	Santa Barbara	Grape	Chardonnay	2
**Je4**	250	*fastidiosa*	CA	Santa Barbara	Grape	Pinot Noir	2
**Je92**	250	*fastidiosa*	CA	Sonoma, MacMurray	Grape	Gruner Veltliner	2
**Je98**	250	*fastidiosa*	CA	Temecula	Grape	N/A	2
**Je100**	250	*fastidiosa*	CA	Temecula	Grape	N/A	2
**16B5**	250	*fastidiosa*	GA	Blackstock, Dahlonega	Grape	Chardonnay	1
**15B1**	250	*fastidiosa*	GA	Blackstock, Dahlonega	Grape	Merlot	1
**16B1**	250	*fastidiosa*	GA	Blackstock, Dahlonega	Grape	Merlot	1
**16M5**	250	*fastidiosa*	GA	Montaluce, Dahlonega	Grape	Pinot Noir	1
**M1**	250	*fastidiosa*	GA	Pierce, Stevie Yong Farm	Blueberry	Meadowlark	10
**VAL VAL 072 Ext**	350	*multiplex*	TX	Del Rio, Val Verde	Giant Ragweed	Ambrosia trifida var. texana	3
**BB08-1**	300	*multiplex*	FL	Palatka, Putnam	Blueberry	Star	4
**AlmaEM3**	300	*multiplex*	GA	Alma	Blueberry	Emerald	1
**S1**	300	*multiplex*	GA	Bacon, Blake Williams Farm	Blueberry	Star	10
**S3**	300	*multiplex*	GA	Bacon, Blake Williams Farm	Blueberry	Star	10
**BBI-64**	300	*multiplex*	GA	N/A	Blueberry	N/A	5

**Georgia Plum**	300	*multiplex*	GA	N/A	Plum	Rosaceae, Prunus sp.	2
**GIL GRA 274 Ext**	300	*multiplex*	TX	Willow City, Gillespie	Annual Sunflower	Asteraceae, Helianthus annuus	3
**R3**	250	*multiplex*	GA	Bacon, Blake Williams Farm	Blueberry	Rebel	10
**S2**	250	*multiplex*	GA	Bacon, Blake Williams Farm	Blueberry	Star	10
**M12**	200	*multiplex*	CA	Kern	Almond	Rosaceae, Prunus dulcis	6
**ESVL**	200	*multiplex*	Spain	Alicante	Almond	N/A	8
**IVIA5901**	200	*multiplex*	Spain	Alicante	Almond	N/A	8
**Oak 92-6**	100	*multiplex*	FL	Palm Beach	Oak	Quercus sp.	4
**De Donno**	350	*pauca*	Italy	Apulia	Olive	Olea europaea L.	9
**Ann-1**	800	*sandyi*	CA	N/A	Oleander	N/A	2
**MED PRI 047**	400	*sandyi*	TX	Median	Oleander	N/A	3

N/A = information non-available

Isolate Sources: 1: Leonardo De La Fuente (Auburn University), 2: Rodrigo Almeida (University of California, Berkeley), 3: Mark Black (Texas A&M University), 4: Donald Hopkins (University of Florida), 5: Harald Scherm (University of Georgia), 6: Jianchi Chen (USDA, Parlier, CA), 7: C.J. Chang (University of Georgia), 8: Blanca Landa (CSIC-IAS, Spain), 9. Maria Saponari (CNR, Italy), 10: Jonathan E. Oliver (University of Georgia, Tifton)
