# Supplementary material for: Phenotypic and Phylogenetic Characterization of Cu Homeostasis among Xylella fastidiosa Strains
Source: Pathogens. 2021 Apr 20;10(4):495. doi: 10.3390/pathogens10040495 (PMC8073393; doi:10.3390/pathogens10040495)
Supplement: Supplementary file 1 [file pathogens-10-00495-s001.zip › pathogens-1184409-supplementary/supp new/Table S2.pdf]

Supplemental Table S2: Information of *X. fastidiosa* isolates and strains for phylogeny.

Strain	Host plant	Place of isolation	Reference	GenBank Accession
Xylella fastidiosa* subsp. *fastidiosa				
**ATCC 35879**	Grape	USA (FL)	[1]	JQAP00000000.1
**CCPM1**	Grape	USA (GA)	[2]	PUJB00000000.1
**CFBP7969**	Grape	USA(NC)	[3]	PHFQ00000000.1
**CFBP7970**	Grape	USA (FL)	[3]	PHFR00000000.1
**CFBP8071**	Almond	USA (CA)	[3]	PHFP00000000.1
**CFBP8073**	Coffee	France	[4]	LKES00000000.1
**CFBP8082**	Annual ragweed	USA (FL)	[3]	PHFT00000000.1
**CFBP8351**	Grape	USA (CA)	[3]	PHFU00000000.1
**DSM 10026**	Grape	USA (FL)	(Varghese JN, unpublished)	FQWN00000000.1
**EB92-1**	Elderberry	USA (FL)	[5]	AFDJ00000000.1
**GB514**	Grape	USA (TX)	[6]	CP002165.1
**gfpWM1-1 Rec1**	Recombinants of WM1-1 with KLN59.3 donor	In vitro	[7]	PUJD00000000.1
**gfpWM1-1 Rec2**	Recombinants of WM1-1 with KLN59.3 donor	In vitro	[7]	PUJE00000000.1
**IVIA5235**	Cherry	Spain	[8]	CP047171.1
**M23**	Almond	USA (CA)	[9]	CP001011.1
**NS1-CmR**	Mutant of TemeculaL	In vitro	[10]	PUJF00000000.1
**NS1pglA Rec**	Recombinant of NS1-CmR and pglA-KmR	In vitro	[11]	PUJG00000000.1
**pglA-KmR**	Grape	USA (CA)	[12]	PUJH00000000.1
**Stag's Leap**	Grape	USA (CA)	[13]	LSMJ00000000.1
**Temecula1**	Grape	USA (CA)	[14]	PUJI00000000
**Temecula1gfp**	Mutant of Temecula1	In vitro	[15]	PUJC00000000.1
**TemeculaL**	Grape	USA (CA)	[16]	PUJJ00000000.1
**TemeculaLAlmaRec1**	Recombinant of TemeculaL with AlmaEM3 donor	In vitro	[16]	PUIW00000000.1
**TemeculaLAlmaRec2**	Recombinant of TemeculaL with AlmaEM3 donor	In vitro	[16]	PUIX00000000.1

**TemeculaStar**	Grape	USA (GA)	[16]	PUJI00000000.1
**TPD3**	Grape	Taiwan	[17]	VJWG00000000.1
**TPD4**	Grape	Taiwan	[17]	VJWH00000000.1
**WM1-1**	Grape	USA (GA)	[2]	PUJK00000000.1
**XYL1732**	Grape	Spain	[18]	QTJT00000000.1
**XYL2055**	Grape	Spain	[18]	QTJS00000000.1
Xylella fastidiosa* subsp. *morus				
**MUL0034**	Mulberry	USA (CA)	[19]	CP006740.1
**Mul-MD**	Mulberry	USA (MD)	[20]	AXDP00000000.1
Xylella fastidiosa* subsp. *sandyi				
**CFBP8356**	Coffee	Costa Rica	[3]	PHFV00000000.1
**Ann-1**	Oleander	USA (CA)	[19]	AAAM00000000.4
**CO33**	Coffee	Italy	[21]	LJZW00000000.1
Xylella fastidiosa* subsp. *multiplex				
**AlmaEM3**	Blueberry (Emerald)	USA (GA)	[22]	PUIY00000000.1
**BB01**	Blueberry	USA (GA)	[23]	MPAZ00000000.1
**BB08-1**	Blueberry	USA (FL)	[22]	PUIZ00000000
**CFBP8078**	Periwinkle	USA (FL)	[3]	PHFS00000000.1
**CFBP8417**	Spanish Broom	France	[3]	LUYB00000000.1
**CFBP8418**	Spanish Broom	France	[3]	LUYA00000000.1
**Dixon**	Almond	USA (CA)	[24]	AAAL00000000.2
**ESVL**	Almond	Spain	[25]	QPQV00000000.1
**IVIA5901**	Almond	Spain	[8]	CP047134.1
**M12**	Almond	USA (CA)	[9]	CP000941
**sycamore Sy-VA**	Sycamore	USA (VA)	[20]	JMHP00000000.1
**TOS14**	Spanish Broom	Italy	[26]	SMTJ00000000.1

**TOS4**	Almond	Italy	[26]	SMTH00000000.1
**TOS5**	Myrtle-leaf milkwort	Italy	[26]	SMTI00000000.1
**ATCC 35871**	Hybrid Plum	USA (GA)	(Kyrpides et al, unpublished)	AUAJ00000000.1
**Griffin-1**	Oak	USA (GA)	[27]	AVGA00000000.1
Xylella fastidiosa **subsp.** ***pauca***				
**3124**	Coffee	Brazil (São Paulo)	[28]	CP009829.1
**CFBP8072**	Coffee	France	[4]	LKDK00000000.1
**CoDiRO**	Olive	Italy (Apulia)	[29]	JUJW00000000.1
**De Donno**	Olive	Italy (Apulia)	[30]	CP020870.1
**PD7202**	Coffee	Netherlands	[31]	RRUA00000000.1
**PD7211**	Coffee	Netherlands	[31]	RRTZ00000000.1
**32**	Coffee	Brazil (São Paulo)	[32]	AWYH00000000.1
**11399**	Orange	Brazil	[33]	JNBT00000000.1
**6c**	Coffee	Brazil (São Paulo)	[32]	AXBS00000000.2
**9a5c**	Sweet orange	Brazil (São Paulo)	[34]	AE003849.1
**COF0324**	Coffee	Brazil	(Knight et al, 2017, unpublished)	LRVG00000000.1
**COF0407**	Coffee	Costa Rica	(Knight et al, 2017, unpublished)	LRVJ00000000.1
**CVC0251**	Sweet orange	Brazil	(Knight et al, 2017, unpublished)	LRVE00000000.1
**CVC0256**	Sweet orange	Brazil	(Knight et al, 2017, unpublished)	LRVF00000000.1
**Fb7**	Sweet orange	Argentina (Corrientes)	[35]	CP010051.2
**Hib4**	Hibiscus	Brazil (São Paulo)	(Pierry and da Silva, 2017, unpublished)	CP009885.1
**J1a12**	Sweet orange	Brazil (São Paulo)	(Pierry and da Silva, 2017, unpublished)	CP009823.1
**OLS0478**	Oleander	Costa Rica	(Knight et al, 2017, unpublished)	LRVI00000000.1
**OLS0479**	Oleander	Costa Rica	(Knight et al, 2017, unpublished)	LRVH00000000.1
**Pr8x**	Plum	Brazil (São Paulo)	(Pierry and da Silva 2015, unpublished)	CP009826.1

**Salento-1**	Olive	Italy (Apulia)	[36]	CP016608.1
**Salento-2**	Olive	Italy (Apulia)	[37]	CP016610.1
**U24D**	Sweet orange	Brazil (São Paulo)	(Da Silva, 2017 unpublished)	CP009790.1

Reference:

1. Chen, J.; O'Leary, M.; Burbank, L.; Zheng, Z.; Deng, X. Whole Genome Sequence of *Xylella fastidiosa* ATCC 35879 T and Detection of Genome Rearrangements Within Subsp. *fastidiosa*. *Curr. Microbiol.* **2020**, *77*, 1858–1863.
2. Denancé, N.; Briand, M.; Gaborieau, R.; Gaillard, S.; Jacques, M.-A. Identification of genetic relationships and subspecies signatures in *Xylella fastidiosa*. *BMC Genom.* **2019**, *20*, 239.
3. Jacques, M.-A.; Denancé, N.; Legendre, B.; Morel, E.; Briand, M.; Mississipi, S.; Durand, K.; Olivier, V.; Portier, P.; Poliakoff, F. New coffee plant-infecting *Xylella fastidiosa* variants derived via homologous recombination. *Appl. Environ. Microbiol.* **2016**, *82*, 1556–1568.
4. Zhang, S.; Flores-Cruz, Z.; Kumar, D.; Chakrabarty, P.; Hopkins, D.L.; Gabriel, D.W. The *Xylella fastidiosa* biocontrol strain EB92-1 genome is very similar and syntenic to Pierce's disease strains. *J. Bacteriol.* **2011**, *193*, 5576–5577.
5. Schreiber, H.; Koirala, M.; Lara, A.; Ojeda, M.; Dowd, S.; Bextine, B.; Morano, L. Unraveling the first *Xylella fastidiosa* subsp. *fastidiosa* genome from Texas. *Southwest. Entomol.* **2010**, *35*, 479–483.
6. Kandel, P.P.; Almeida, R.P.; Cobine, P.A.; De La Fuente, L. Natural competence rates are variable among *Xylella fastidiosa* strains and homologous recombination occurs in vitro between subspecies fastidiosa and multiplex. *Mol. Plant Microbe Interact.* **2017**, *30*, 589–600.
7. Landa, B.B.; Velasco-Amo, M.P.; Marco-Noales, E.; Olmo, D.; López, M.M.; Navarro, I.; Monterde, A.; Barbé, S.; Montes-Borrego, M.; Román-Écija, M. Draft genome sequence of *Xylella fastidiosa* subsp. *fastidiosa* strain IVIA5235, isolated from Prunus avium in Mallorca Island, Spain. *Microbiol. Resour. Announc.* **2018**, *7*, e01222-18.
8. Chen, J.; Xie, G.; Han, S.; Chertkov, O.; Sims, D.; Civerolo, E. Whole genome sequences of two *Xylella fastidiosa* strains (M12 and M23) causing almond leaf scorch disease in California. *J. Bacteriol.* **2010**, *192*, 4534–4534.
9. Matsumoto, A.; Young, G.M.; Igo, M.M. Chromosome-based genetic complementation system for *Xylella fastidiosa*. *Appl. Environ. Microbiol.* **2009**, *75*, 1679–1687.
10. Kandel, P.P.; Lopez, S.M.; Almeida, R.P.; De La Fuente, L. Natural Competence of *Xylella fastidiosa* Occurs at a High Frequency Inside Microfluidic Chambers Mimicking the Bacterium's Natural Habitats. *Appl. Environ. Microbiol.* **2016**, *82*, 5269–5277, doi:10.1128/AEM.01412-16.
11. Roper, M.C.; Greve, L.C.; Warren, J.G.; Labavitch, J.M.; Kirkpatrick, B.C. *Xylella fastidiosa* requires polygalacturonase for colonization and pathogenicity in *Vitis vinifera* grapevines. *Mol. Plant Microbe Interact.* **2007**, *20*, 411–419.
12. Chen, J.; Wu, F.; Zheng, Z.; Deng, X.; Burbank, L.; Stenger, D. Draft genome sequence of *Xylella fastidiosa* subsp. *fastidiosa* strain Stag's leap. *Genome Announc.* **2016**, *4*, doi:10.1128/genomeA.00240-16
13. Van Sluys, M.; De Oliveira, M.; Monteiro-Vitorello, C.; Miyaki, C.; Furlan, L.; Camargo, L.; Da Silva, A.; Moon, D.; Takita, M.; Lemos, E. Comparative analyses of the complete genome sequences of Pierce's disease and citrus variegated chlorosis strains of *Xylella fastidiosa*. *J. Bacteriol.* **2003**, *185*, 1018–1026.

14. Newman, K.L.; Almeida, R.P.P.; Purcell, A.H.; Lindow, S.E. Use of a green fluorescent strain for analysis of *Xylella fastidiosa* colonization of *Vitis vinifera*. *Appl. Environ. Microbiol.* **2003**, *69*, 7319–7327.
15. Potnis, N.; Kandel, P.P.; Merfa, M.V.; Retchless, A.C.; Parker, J.K.; Stenger, D.C.; Almeida, R.P.; Bergsma-Vlami, M.; Westenberg, M.; Cobine, P.A. Patterns of inter-and intrasubspecific homologous recombination inform eco-evolutionary dynamics of *Xylella fastidiosa*. *ISME J.* **2019**, *13*, 2319–2333.
16. Castillo, A.I.; Tuan, S.-J.; Retchless, A.C.; Hu, F.-T.; Chang, H.-Y.; Almeida, R.P. Draft Whole-Genome Sequences of *Xylella fastidiosa* subsp. *fastidiosa* Strains TPD3 and TPD4, Isolated from Grapevines in Hou-li, Taiwan. *Microbiol. Resour. Announc.* **2019**, *8*, doi:10.1128/MRA.00835-19.
17. Gomila, M.; Moralejo, E.; Busquets, A.; Segui, G.; Olmo, D.; Nieto, A.; Juan, A.; Lalucat, J. Draft genome resources of two strains of *Xylella fastidiosa* XYL1732/17 and XYL2055/17 isolated from Mallorca vineyards. *Phytopathology* **2019**, *109*, 222–224.
18. Schuenzel, E.L.; Scally, M.; Stouthamer, R.; Nunney, L. A multigene phylogenetic study of clonal diversity and divergence in North American strains of the plant pathogen *Xylella fastidiosa*. *Appl. Environ. Microbiol.* **2005**, *71*, 3832–3839.
19. Guan, W.; Shao, J.; Davis, R.E.; Zhao, T.; Huang, Q. Genome sequence of a *Xylella fastidiosa* strain causing sycamore leaf scorch disease in Virginia. *Genome Announc.* **2014**, *2*, doi:10.1128/genomea.00773-14.
20. Giampetruzzi, A.; Loconsole, G.; Boscia, D.; Calzolari, A.; Chiumenti, M.; Martelli, G.P.; Saldarelli, P.; Almeida, R.P.; Saponari, M. Draft genome sequence of CO33, a coffee-infecting isolate of Xylella fastidiosa. *Genome Announc.* **2015**, *3*, doi:10.1128/genomea.01472-15.
21. Oliver, J.E.; Sefick, S.A.; Parker, J.K.; Arnold, T.; Cobine, P.A.; De La Fuente, L. Ionome changes in *Xylella fastidiosa*–infected *Nicotiana tabacum* correlate with virulence and discriminate between subspecies of bacterial Isolates. *Mol. Plant Microbe Interact.* **2014**, *27*, 1048–1058, doi:10.1094/MPMI-05-14-0151-R.
22. Van Horn, C.; Chang, C.-J.; Chen, J. De novo whole-genome sequence of *Xylella fastidiosa* subsp. *multiplex* strain BB01 isolated from a blueberry in Georgia, USA. *Genome Announc.* **2017**, *5*, doi:10.1128/genomea.01598-16.
23. Bhattacharyya, A.; Stilwagen, S.; Reznik, G.; Feil, H.; Feil, W.S.; Anderson, I.; Bernal, A.; D'Souza, M.; Ivanova, N.; Kapatral, V. Draft sequencing and comparative genomics of *Xylella fastidiosa* strains reveal novel biological insights. *Genome Res.* **2002**, *12*, 1556–1563.
24. Giampetruzzi, A.; Velasco-Amo, M.P.; Marco-Noales, E.; Montes-Borrego, M.; Roman-Ecija, M.; Navarro, I.; Monterde, A.; Barbé, S.; Almeida, R.P.; Saldarelli, P. Draft genome resources of two strains ("ESVL" and "IVIA5901") of *Xylella fastidiosa* associated with almond leaf scorch disease in Alicante, Spain. *Phytopathology* **2019**, *109*, 219–221.
25. Giampetruzzi, A.; D'Attoma, G.; Zicca, S.; Abou Kubaa, R.; Rizzo, D.; Boscia, D.; Saldarelli, P.; Saponari, M. Draft genome sequence resources of three strains (TOS4, TOS5, and TOS14) of *Xylella fastidiosa* infecting different host plants in the newly discovered outbreak in Tuscany, Italy. *Phytopathology* **2019**, *109*, 1516–1518.
26. Chen, J.; Huang, H.; Chang, C.-J.; Stenger, D.C. Draft genome sequence of *Xylella fastidiosa* subsp. *multiplex* strain Griffin-1 from Quercus rubra in Georgia. *Genome Announc.* **2013**, *1*, doi:10.1128/genomea.00756-13.
27. Li, W.-B.; Pria Jr, W.; Teixeira, D.; Miranda, V.; Ayres, A.; Franco, C.; Costa, M.; He, C.-X.; Costa, P.; Hartung, J. Coffee leaf scorch caused by a strain of *Xylella fastidiosa* from citrus. *Plant Dis.* **2001**, *85*, 501–505.

28. Giampetruzzi, A.; Chiumenti, M.; Saponari, M.; Donvito, G.; Italiano, A.; Loconsole, G.; Boscia, D.; Cariddi, C.; Martelli, G.P.; Saldarelli, P. Draft genome sequence of the *Xylella fastidiosa* CoDiRO strain. *Genome Announc.* **2015**, *3*, doi:10.1128/genomea.01538-14.
29. Giampetruzzi, A.; Saponari, M.; Almeida, R.P.; Essakhi, S.; Boscia, D.; Loconsole, G.; Saldarelli, P. Complete genome sequence of the olive-infecting strain *Xylella fastidiosa* subsp. *pauca* De Donno. *Genome Announc.* **2017**, *5*, doi:10.1128/genomea.00569-17.
30. Bergsma-Vlami, M.; van de Bilt, J.; Tjou-Tam-Sin, N.; Helderman, C.; Gorkink-Smits, P.; Landman, N.; van Nieuwburg, J.; van Veen, E.; Westenberg, M. Assessment of the genetic diversity of *Xylella fastidiosa* in imported ornamental Coffea arabica plants. *Plant Pathol.* **2017**, *66*, 1065–1074.
31. Grisard, E.C.; Teixeira, S.M.R.; de Almeida, L.G.P.; Stoco, P.H.; Gerber, A.L.; Talavera-López, C.; Lima, O.C.; Andersson, B.; de Vasconcelos, A.T.R. Trypanosoma cruzi clone Dm28c draft genome sequence. *Genome Announc.* **2014**, *2*, doi:10.1128/genomea.01114-13.
32. Niza, B.; Merfa, M.V.; Alencar, V.C.; Menegidio, F.B.; Nunes, L.R.; Machado, M.A.; Takita, M.A.; de Souza, A.A. Draft genome sequence of 11399, a transformable citrus-pathogenic strain of *Xylella fastidiosa*. *Genome Announc.* **2016**, *4*, doi:10.1128/genomea.01124-16.
33. Simpson, A.J.G.; Reinach, F.d.C.; Arruda, P.; Abreu, F.A.d.; Acencio, M.; Alvarenga, R.; Alves, L.C.; Araya, J.E.; Baia, G.S.; Baptista, C. The genome sequence of the plant pathogen *Xylella fastidiosa*. *Nature* **2000**, *406*, 151–157.
34. da Silva, V.S.; Shida, C.S.; Rodrigues, F.B.; Ribeiro, D.C.; de Souza, A.A.; Coletta-Filho, H.D.; Machado, M.A.; Nunes, L.R.; de Oliveira, R.C. Comparative genomic characterization of citrus-associated *Xylella fastidiosa* strains. *BMC Genom.* **2007**, *8*, 474.
35. Bleve, G.; Marchi, G.; Ranaldi, F.; Gallo, A.; Cimaglia, F.; FRANCESCO LOGRIECO, A.; Mita, G.; Ristori, J.; Surico, G. Molecular characteristics of a strain (Salento-1) of *Xylella fastidiosa* isolated in Apulia (Italy) from an olive plant with the quick decline syndrome. *Phytopathol. Mediterr.* **2016**, *55*, 139–146.
36. Ramazzotti, M.; Cimaglia, F.; Gallo, A.; Ranaldi, F.; Surico, G.; Giovanni, M.; Bleve, G.; Marchi, G. Insights on a founder effect: The case of *Xylella fastidiosa* in the Salento area of Apulia, Italy. *Phytopathol. Mediterr.* **2018**, *57*, 8–25.
